# Temperature-Compensated Force/Pressure Sensor Based on Multi-Walled Carbon Nanotube Epoxy Composites

**DOI:** 10.3390/s150511133

**Published:** 2015-05-12

**Authors:** Nghia Trong Dinh, Olfa Kanoun

**Affiliations:** Electrical Measurements and Sensor Technology, Technische Universität Chemnitz, Reichenhainer Str. 70, Chemnitz 09126, Germany; E-Mail: dinh_trong_nghia@hotmail.com

**Keywords:** carbon nanotubes, multi-walled carbon nanotubes, epoxy, composite, force sensor, pressure sensor, electrical response, thermal response, temperature compensation

## Abstract

In this study, we propose a multi-walled carbon nanotube epoxy composite sensor for force and pressure sensing in the range of 50 N–2 kN. A manufacturing procedure, including material preparation and deposition techniques, is proposed. The electrode dimensions and the layer thickness were optimized by the finite element method. Temperature compensation is realized by four nanocomposites elements, where only two elements are exposed to the measurand. In order to investigate the influence of the filler contents, samples with different compositions were prepared and investigated. Additionally, the specimens are characterized by cyclical and stepped force/pressure loads or at defined temperatures. The results show that the choice of the filler content should meet a compromise between sensitivity, temperature influence and noise behavior. At constant temperature, a force of at least 50 N can be resolved. The measurement error due to the temperature influence is 150 N in a temperature range of −20°C−50°C.

## 1. Introduction

The measurement of mechanical quantities, such as force, torque or pressure, is a challenging task in science and engineering. Very often, a deformation element, like a beam, a membrane or an axle [1], is needed, so that the force, pressure or torque is transformed to a strain, which can be measured by a strain sensor. For this, metallic or semiconductor strain gauges (SGs) can be used. Due to lower signal noise and higher mechanical stability, metallic SGs are more frequently used than semiconductor SGs. Nevertheless, metallic SGs show a limitation considering their gauge factor in the range of two, which is comparatively low. New promising sensors based on polymer [2], printable metal inks [3], inorganic [4] or organic composites [5] show a higher sensitivity than metallic SGs and provide therefore interesting solutions for many applications. Recently, many researchers reported on flexible electronics [6], flexible strain sensors [7] and pressure sensors [8] by using nanocomposites. Most of them use nanoparticles, such as quantum dot, nanowire, graphene and carbon nanotubes (CNTs). The one-dimensional structure of CNTs and their semiconducting or quasi-metallic properties [9,10] make them suitable for a large field of applications. The performances of a single CNT referring to the electrical transport [11], the thermal transport [12], in electronic applications [13] and also the sensitivity in sensor application, e.g., for strain measurement [14,15] are better than CNT networks. Even though a single CNT has better performance, there are enormous technological challenges for series fabrication. An additional advantage of the CNTs in comparison to other nanomaterials is the high aspect ratio between length and diameter; that is why the electrical percolation threshold of CNTs is three-times lower than carbon black in Zhao's report [16]. Diverse deposition methods can be applied to fabricate a piezoresistive film, such as spraying [17], filtering [18], spin coating [19] dip coating [20], inkjet printing [21] and screen printing techniques [22].

The piezoresistivity of nanomaterials in polydimethylsiloxane (PDMS) for pressure application are investigated in previous reports, e.g., for carbon black [23] and carbon nanotubes [24]. However, the applicable pressure is low (0–0.2 MPa) [24] for elastomer. This measurement range is interesting, for example, on the human periphery [25].

For a higher pressure range, for example, in machines, polymers with a higher elasticity modulus, such as thermoplastic or thermosetting plastic, are necessary. The strain dependency of CNT composites in such materials was investigated for different material compositions. The gauge factor decreases by increasing the CNT filler content [26]. However, Hu *et al.* show that the electrical signal of low filler content could be unstable (noisy) due to the unstable destruction and construction of conductive paths [5]. Kang *et al.* show a repeatable electrical signal and fast sensor response time in less than 0.3ms for 0.05 wt% single-walled carbon nanotubes (SWCNTs), which are embedded in polyimide, in a pressure measurement up to 5 MPa [27]. However, the signal quality depending on different filler content was not investigated. Mohiuddin *et al.* dispersed multi-walled carbon nanotubes (MWCNTs) in polyether ether ketone. In this investigation, the temperature dependence from 20 °C up to 140 °C and the pressure dependence up to 40 MPa were studied [28]. However, the electrical responses show a big drift after temperature treatment and pressure loading [29].

In this paper, we investigate the sensing behavior of MWCNT-epoxy composite by applying pressure with different loading profiles. Thereby, not only the high sensitivity is important, also the sensitivity to temperature influence and the signal-to-noise ratio are further important criteria. For these reasons, another focus of this work is the comparison between the pressure sensitivity, the temperature influence and the signal noise of the MWCNT-epoxy composite by varied filler contents. The resistance of a CNT composite is strongly dependent on the temperature [30,31]; this effect reduces the measurement accuracy and should be therefore compensated. Hence, we investigate the efficiency of the temperature compensation with a Wheatstone bridge.

## 2. Preparation of the Nanocomposite

The shell quality and the chirality of the CNTs play a major roll in the conductivity of a composite. Another aspect is the load transfer between the polymer matrix and the CNTs. Due to the covalent bonding between functionalized CNTs and the polymer matrix, the elastic modulus with functionalized CNTs is higher than non-functionalized CNTs [32]. However, the functionalization can destroy the CNT shells and reduces the conductivity of the composite [33]. For this reason, non-functionalized MWCNTs are chosen in this study.

The fabrication parameters and the dispersion methods have a big influence to the mechanical and electrical properties [34,35]. A high shear rate can shorten the CNTs and decreases the conductivity [34]. Therefore, the fabrication parameters need to be chosen carefully. Another effect is the rise in viscosity after MWCNTs are added into the epoxy [36]. Too high a viscosity makes the deposition of homogenous and reproducible layers difficult.

### 2.1. Characterization of MWCNTs

**Figure 1:**
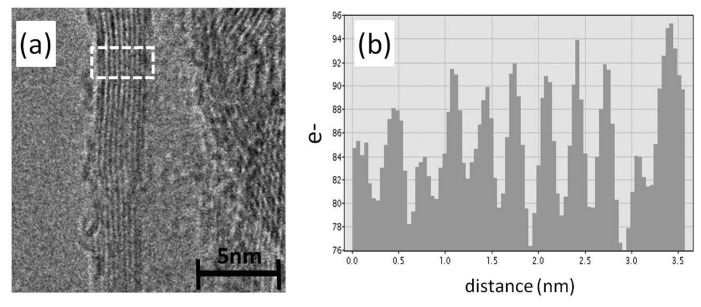
Analysis of MWCNTs by HRTEM; (**a**) HRTEM image of MWCNT; (**b**) analysis of the shell numbers.

The outer shells are the shells mainly responsible for the electron transport in MWCNTs [37,38]. An exchange of electrons between two perfect shells is unusual, because the resistance between the shells should be 10,000-times [39] higher than in one shell. Subsequent investigations point out that defects increase the resistance, but controlled shell defects can reduce the distance between two shells from 0.34 nm–0.138 nm [40] and increase the number of conduction channels. Agrawal *et al.* demonstrated that a moderate shell defect has even higher conductivity than a MWCNT with less shell defects [41]. The conductivity of a MWCNT depends also on the number of the conduction channels and, consequently, the MWCNT diameter [42]. Therefore, the shell quality and the diameter of the commercially available MWCNTs (purchased from Future Carbon) are investigated by high resolution transmission electron microscopy (HRTEM). Except for few positions, most MWCNTs have continuous shells with an average diameter of 11nm. The image of one MWCNT is depicted in Figure 1a. The corresponding average shell numbers are eleven (Figure 1b).

### 2.2. MWCNT-Epoxy Composite

**Figure 2:**
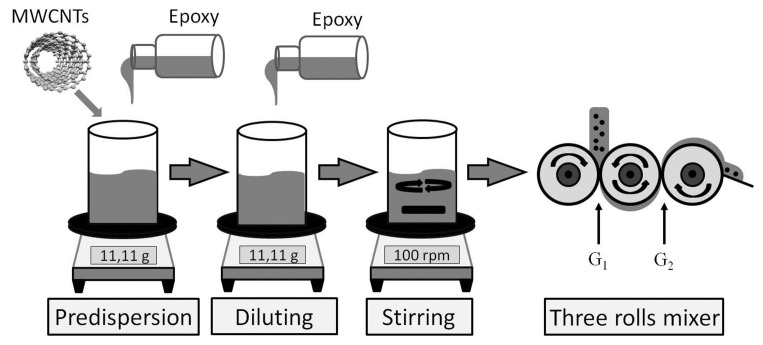
Preparation processes of the MWCNT-epoxy composite.

In this study, composites with MWCNT filler contents from 1 wt% up to 2wt% are investigated. The whole preparation process of the MWCNT-epoxy composite are depicted in Figure 2. The MWCNTs and the epoxy, namely Rütapox^®^L20 (purchased from R & G Faserverbundwerkstoffe GmbH), are pre-dispersed by “Future Carbon” with a filler content of 4 wt%. Afterwards, the dispersion was diluted to the desired concentration. After the dispersion was diluted, the dispersions were mixed with a magnetic stirrer at 100 rpm for 6 h. The samples are immersed in heated silicon oil at 80 °C to reduce the viscosity during this process. To achieve more homogeneity, the dispersions are subjected to a three-roll mixer from the company EXAKT with the name E80. The MWCNT composite is dispersed three times with different gap spacing, while the velocity with 50 rpm remains the same. In the first dispersion step, the gap space between the first and the second rolls (G1) is 50 μm, and the gap space between the second and the third rolls (G2) is 20 μm. In the second step, the gap spaces are 20 μm (G1) and 10 μm (G2). Finally, in the third step, the gap spaces are 10 μm (G1) and 5 μm (G2).

## 3. Device Design and Layer Deposition

In order to improve the pressure sensitivity, we propose to use a Wheatstone bridge, which is also suitable for reducing the temperature effects. Due to the low conductivity of the composite, an interdigital electrode was chosen. The electrodes of an interdigital structure are parallel interconnected, so that the total resistance is reduced. Additionally, the influences of the electrode dimensions and the layer thickness to the nominal resistance are investigated by the finite element method (FEM).

### 3.1. Sensing Structures and Temperature Compensation Concept

**Figure 3:**
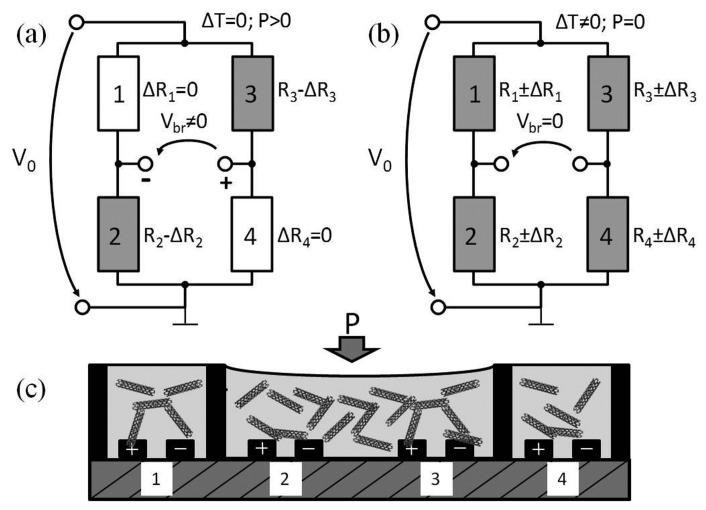
Sensing concept with a half Wheatstone bridge: (**a**) half bridge circuit for the force/pressure sensing; (**b**) full bridge circuit for the temperature compensation; (**c**) four sensing elements on a substrate.

The resistance changes of the realized sensing elements will have the same sign, if they are pressed at the same time. Therefore, we propose to use the middle elements in a full bridge for the force/pressure measurement. This corresponds to a half bridge structure (Figure 3a,c). Elements 1 and 4 are only responsible for the balance of the bridge voltage (Vbr). They have the same temperature, like Elements 2 and 3, but are not exposed to the deformation. When a pressure is applied to Elements 2 and 3, the resistance will be reduced (−Δ*R*_2_; −Δ*R*_3_). The resistance changes cause potential changes of the voltages; hence, the bridge voltage is unequally zero. In case of a half bridge, the resistance change Δ*R* (for Δ*R* = Δ*R*_2_ = Δ*R*_3_) can approximately be calculated with following equation, whereby *V*_0_ is the supply voltage, *V_br_* the bridge voltage and *R_0_* is a referent resistance at a defined force.

(1) $$\Delta R=\frac{V_{br}\cdot 2\cdot R_{0}}{V_{0}}$$

For the temperature compensation, all four elements are exposed to the same temperature (Figure 3b). If the nominal resistances and temperature coefficients of all four elements are equal, the resistance changes due to temperature have the same sign and value (that means Δ*R1* = Δ*R*_2_ = Δ*R*_3_ = Δ*R*_4_). In this case, the contribution of temperature change to the bridge voltage is neglectable, so that the temperature influence is compensated.

### 3.2. Electrode Modeling and Design

The resistances of the sensing elements depend on the conductivity of the composite, electrode width, electrode distance between two electrodes and the thickness of the layer. Depending on the filler content, shell quality, MWCNT diameter and length, the conductivity of MWCNT composites can be distributed in a big range from 2 × 10^−4^ S/m up to 1 × 10^4^ S/m [43–48]. For this reason, a quantitative resistance calculation is not appropriate, due to the unknown conductivity. For temperature compensation within a Wheatstone bridge, elements should have similar resistance values. If the four resistances have a big variation, the temperature compensation will not be optimal. The resistance values especially depend on the manufacturing tolerances of the electrodes and the layer thickness. That is why we investigate the resistance changes due to the changing of these parameters by FEM in this section, in order to find out the suitable electrode dimensions and layer thicknesses. That led to less dependence of the nominal resistance due to the manufacturing tolerances.

**Figure 4:**
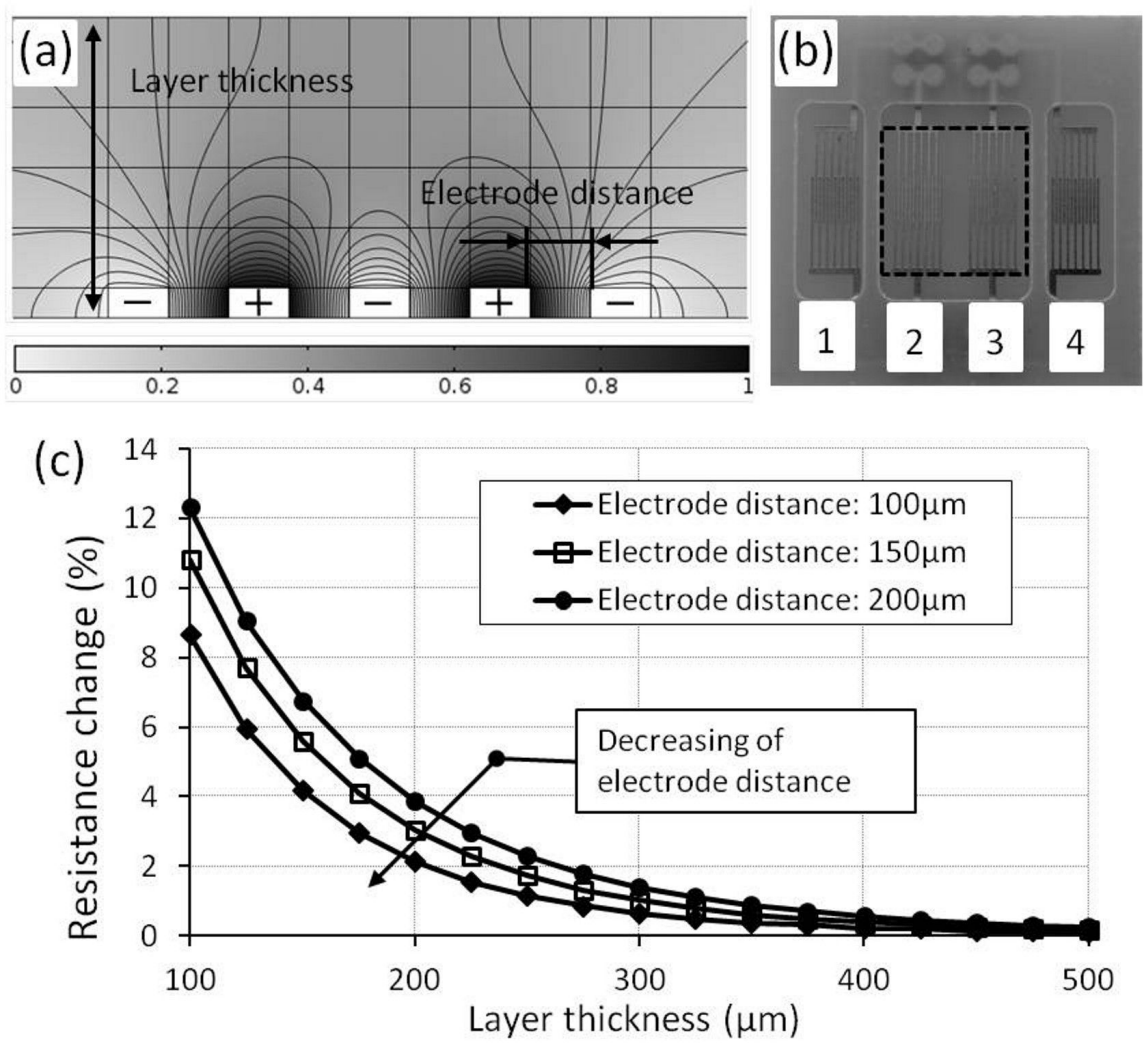
Electrode modeling and design: (**a**) electrical field distribution between interdigital electrodes; (**b**) image of fabricated interdigital electrodes on printed circuit board substrate; (**c**) resistance change depends on the layer thickness and electrode distance.

The FEM modeling was carried out by using the software, Comsol Multiphysics^®^ (Version 12), with the “AC/DC Module” and the “Electric Currents Interface”. The model was simulated in a stationary electrical field, and a reduced 3D model was used to decrease the computing time. As model inputs, the material copper with a conductivity of 5.99×10^7^ S/m for the electrodes and a representative conductivity of 1 S/m for the MWCNT composite was chosen. As boundary conditions, a current input of 1 mA on the plus electrodes was defined, and the minus electrodes were grounded. A systematic distribution of the electrical field in the interdigital electrodes is shown in Figure 4a.

By varying the electrode distance (100 μm–200 μm) and layer thickness (100 μm–500 μm), the resistance of the element was calculated by simulation. The results of the model show that the resistance change is reduced by increasing the layer thickness, and it reaches saturation at a layer thickness of 400 μm. By decreasing the electrode distance between two electrodes, saturation is already reached for the thinner layer (Figure 4c). In other words, to achieve similar resistance values, the electrode distance should be as small as possible and the layer thickness as thick as possible.

Depending on the fabrication limit, the optimized dimensions of 150 μm for the electrode width and 100 μm for the distance between two electrodes were chosen. To get a high reproducibility (variation of less than 1%) of the nominal resistance, the layer thickness should be greater than 400 μm. Based on these modeling results, interdigital electrodes with four elements were designed and fabricated on conventional printed circuit board substrate (FR-4) (Figure 4b).

### 3.3. Piezo-Sensitive Layer Deposition

To have reproducible resistance, the layer thickness should be greater than 400 μm. For such a thickness, screen printing or blade coating are suitable techniques. In this work, blade coating is used, due to the lower demand on the morphology of the dispersion. Before the deposition process, the dispersed composite was mixed with the curing agent, namely epoxy hardener (EPH) 161 (purchased from R & G Faserverbundwerkstoffe GmbH) at a ratio of 4:1.

**Figure 5:**
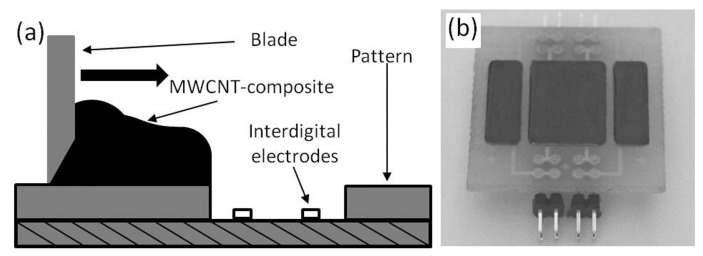
Fabrication of the piezo-sensitive layer: (**a**) principle of layer deposition by blade coating; (**b**) image of the fabricated sensing device.

After the blade coating, the composite was dried at room temperature for 24 h. Afterwards, an additional annealing process at 60 °C for a further 24 h was applied to achieve the final mechanical strength. The principles of the layer deposition and the fabricated sensing device are depicted in Figure 5.

## 4. Force/Pressure and Temperature Measurement Setups

**Figure 6:**
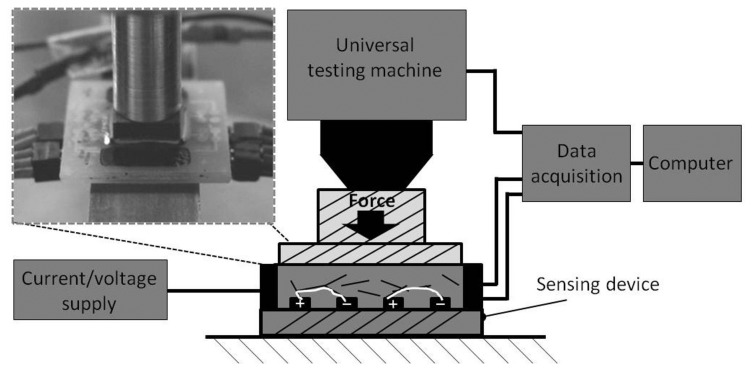
Force/pressure measurement setup.

In order to evaluate the reproducibility of the sensing response and the signal-to-noise ratio, the defined pressure load and temperature are needed. Thereby, a synchronization between the excitation signals and response signal is necessary. Additionally, the measurement setups should be able to generate static, cyclical and stepped loads. Furthermore, the sensing elements can be characterized separately (one element) or in a Wheatstone bridge (four elements). Two different measurement setups, namely pressure and temperature measurements, were used in this study. In Figure 6, the scheme of the pressure measurement is pictured.

A source-meter (Keithley 2026) is responsible for the power supply. When one single element is measured, a constant current of 15 μA is applied. For the bridge circuit, a supply voltage of 0.2 V was chosen. The resistance change can be calculated by the measurement of the bridge voltage. Using the data acquisition device (National Instruments 9219), the bridge voltage and the force signal from the universal testing machine are measured. The universal testing machine presses the sensitive area through a stamp of 1 cm^2^ up to a force of 2 kN (20 MPa). Thereby, a velocity of 0.1 mm/min was chosen. All signals are recorded from the program “Labview” for subsequent analysis. The temperature measurement setup has nearly the same configuration. Instead of the universal testing machine, a climate chamber (Vötsch VT 4002) is used. The samples are measured from −20 °C up to 80 °C. To make sure that the samples have the desired temperature, the temperature change is 5 °C and remains constant for 30 min.

## 5. Results and Discussions

A suitable viscosity of the composite is very important for a homogenous layer deposition. Therefore, the viscosity of the MWCNT-epoxy composite is measured in this section. In order to investigate the influence of the filler content, the sensitivity and the signal-to-noise ratio from different MWCNT concentrations are characterized. For a sensor application, the temperature influences is not negligible. A comparison between the resistance change due to the pressure and the cross-sensitivity due to the temperature can give an assessment of the resolution or the working temperature. To compensate for the temperature influence, a bridge circuit was used. Finally, the temperature sensitivity of one element and the temperature sensitivity of the bridge circuit are compared.

### 5.1. Viscosities of MWCNT-Epoxy Dispersion

**Figure 7:**
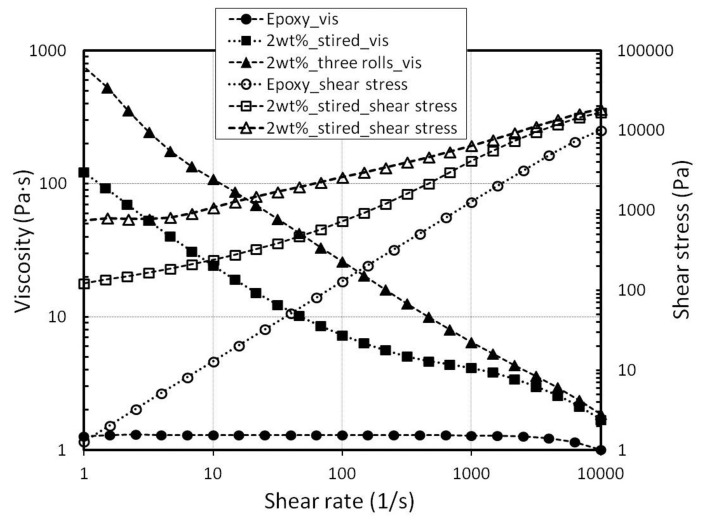
Viscosity of the dispersions at different mixing states.

Bauhofer *et al.* show a relationship between the rheology and the conductivity of an MWCNT-epoxy [44]. The viscosity strongly increases after the electrical percolation threshold is reached. Furthermore, the suitable rheology is important to get homogeneous layers by the blade coating process. On that score, the rheology is measured with a rheometer “Physica MCR 301, Anton Paar”. Figure 7 shows the viscosity and the shear rate of the unfilled epoxy and 2 wt%-filled dispersion of different mixing states.

The unfilled epoxy has a viscosity of 1.3 Pa·s and shows the behavior of a Newtonian fluid, where the viscosity is independent of the shear rate. On the other hand, 2 wt%-filled epoxy shows a shear-thinning behavior, and the viscosity is multiply increased. Additionally, a strong increase of viscosity was obtained after the three-roll mixing process. The viscosity of a 2 wt% magnet-stirred dispersion is 122 Pa·s; however the viscosity of the same filler content fabricated by the three-roll mixer is 794 Pa·s (at a shear rate of 1 (1/s)). The high viscosity can be explained by a more effective unbundling of MWCNTs through the three-roll mixer. Another possible reason is the alignment of MWCNTs caused by the mixing process [49]. To compare the dispersion efficiency, samples with different mixing methods are fabricated by blade coating (Figure 5a). The samples with 2 wt% fabricated by a magnetic stirrer have an average resistance of 3.64 kΩ (at 20 °C). Samples fabricated by the additional mixing step (three-roll mixer) have an average value of 3.1 kΩ (at 20 °C). The nominal resistance after the three-roll mixing process is decreased by 15%. This result leads to the conclusion that the MWCNTs are more effective when unbundled by the three-roll mixer.

### 5.2. Resistance Behavior under Force/Pressure Loads

**Figure 8:**
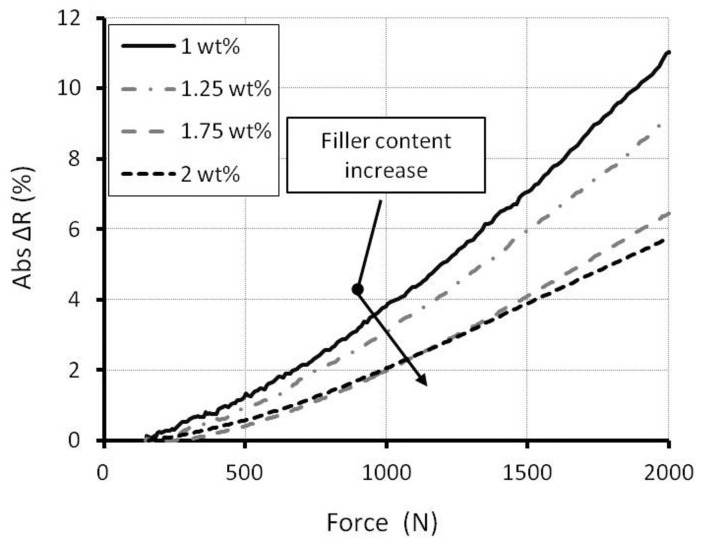
Absolute resistance change from different filler contents.

In order to investigate the sensitivity depending on the filler content, only the resistance of a single element is measured. By applying a constant current of 15 μA, the voltage drop is measured, and the resistance is calculated by Ohm's law. Due to the clearance in the measurement setup, an initial load is necessary to press every component together. A stable electrical response is measured after an initial load between 50 and 150 N has been applied. The resistance decreases when the force is applied. Thus, this indicates that the establishment of electrical paths is dominating. For better comparisons, the absolute resistance changes (Abs ΔR) were calculated. The absolute resistance change at different filler contents is shown in Figure 8.

All curves show a non-linear behavior between force and resistance changes. Samples with lower filler content have higher sensitivity. This result corresponds with the experimental result of Yin *et al.* on the bending beam [43] and the numerical modeling result of Hu *et al.* [48]. The resistance change can be explained by the percolation theory [50] and the tunneling effect [48]. In a CNT network, the total resistance change is induced by the change of the number of the contact resistances and the tunnel resistances. At lower filler content, both of these resistance components are more marked [5]. For that reason, lower filler content has a higher sensitivity.

**Figure 9:**
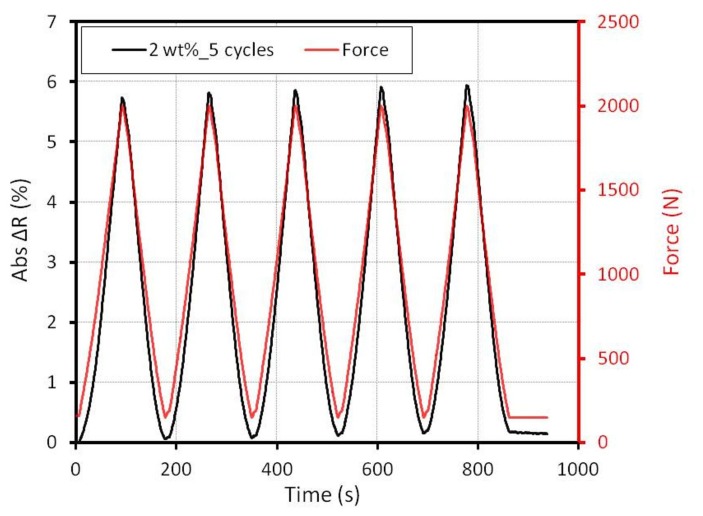
Absolute resistance change at five loading cycles (sample with 2wt%).

To explore the repeatability of the electrical signal, an alternating force from 150 N up to 2 kN was applied to the sample. With a velocity of 0.1 mm/min, the electrical signal of five cycles was measured. The absolute resistance change of a sample with 2 wt% is depicted in Figure 9.

A small drift was obtained. Especially between the first cycles and the second cycle, there is a distinct difference on the reversal point. Nonetheless, the electrical response shows a reproducible characteristic in the following cycles. Before and after the measurement, the resistance of the sample was measured. The current-voltage (I-V) curves show no identifiable difference. Hence, we conclude that the stability of the MWCNT-epoxy is given up to a force of 2 kN (pressure 20 MPa). There is no delay in the electrical response obtained. In one second, two measurements are taken (2 Hz). For that reason, a response time under 0.5 s can be expected.

Compared to the results from Yin *et al.* with MWCNT-epoxy [43] and Mohiuddin *et al.* with MWCNTs in polyether ether ketone (PEEK) [29], this performance shows a better reproducibility. The reproducibility in this study is comparable with the result of Kang *et al.*, who used a SWCNT/polyimide composite [27] in pressure measurement. In contrast to Kang's report, less expensive MWCNTs were used in this work, and the sensitivity is higher. Furthermore, the applied pressure on the composite is up to 20 MPa, whereby the maximum pressure in the work of Kang *et al.* is 5MPa.

**Figure 10:**
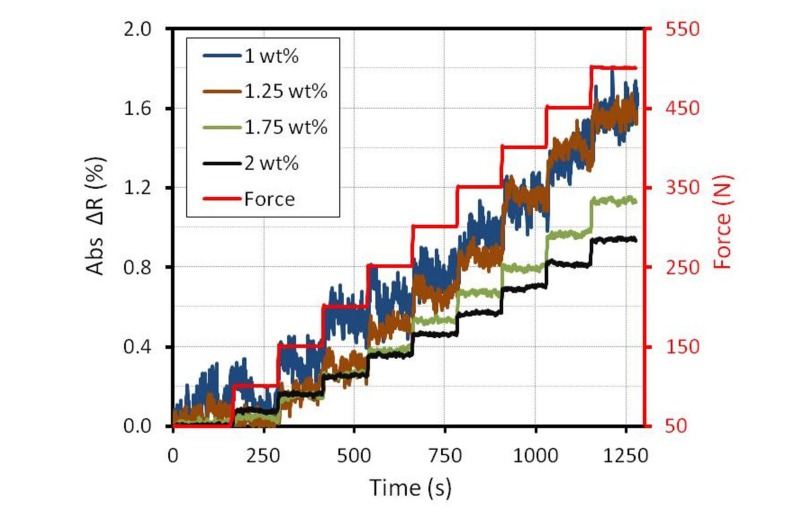
Stability of the signals by stepped loading in a half bridge circuit.

In the next step, the four elements are collected into a Wheatstone bridge (sensor structure). Only the two elements in the middle are pressed by the testing machine. The measurement was started by an initial load of 50 N. To investigated the stability of the electrical response, the forces are increased in 50-N (0.5 MPa) steps. In each step, the force remained constant for two minutes. For a half bridge, the resistance change was calculated by Equation (1). The absolute resistance changes for different filler content by the stepped loading are depicted in Figure 10.

The signal of the 1.75 wt% and 2 wt% samples follow the loading. However, the signal of the 1 wt% sample could not follow the stepped loading due to the noise. Close to the electrical percolation threshold, the construction and destruction of electrical paths are unbalanced. In a small strain area, the resistance can take turns increasing or decreasing, although the total strain increases. Hence, the resistance change is unstable. This result corresponds with the theoretical and experimental result of Hu *et al.* for the stretching case [5]. The result of this work can show the noise behavior for lower filler content also for the compression case.

### 5.3. Temperature Influence on One Element

**Figure 11:**
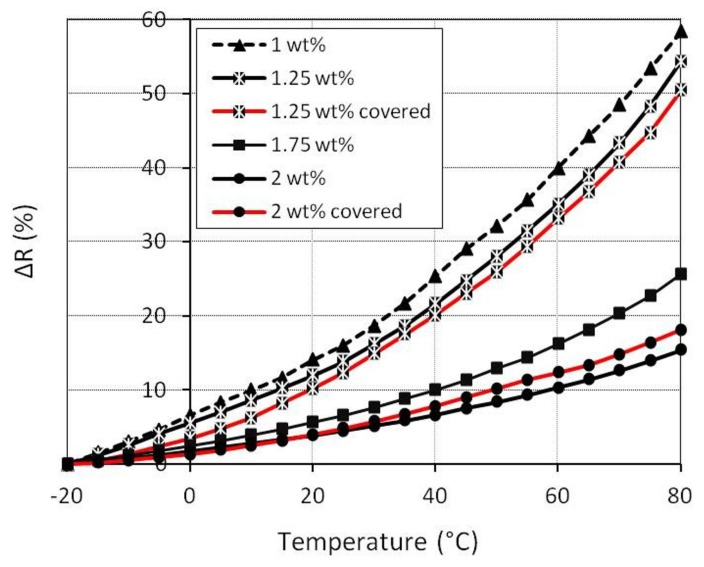
Temperature influence on the resistance. Black lines indicate native MWCNT specimens; red lines indicate PDMS-covered MWCNT specimens.

As mentioned before, only one element was measured to investigate the temperature influence. The measurement begins at 80 °C. Before the measurement starts, the sample was warmed up for 6 h at 80 °C. The temperature decreased in 5 °C steps and was maintained constant for 30 min. After the desired temperature was reached, the resistance was measured ten times, and the average value was calculated. The reference resistances for this experiment are at −20 °C. First of all, native MWCNT specimens were measured, as indicated with black lines in Figure 11. In order to avoid the humidity influence, specimens with 1.25 wt% and 2 wt% were covered with a 2-mm layer of polydimethylsiloxane (PDMS) afterwards. The resistances depending on the temperature of the covered specimens are indicated with red lines in Figure 11.

Lower filler content shows a higher sensitivity towards the temperature, and all samples have a positive temperature coefficient. This behavior is inverse to the negative temperature coefficient of MWCNTs, which are aligned and interconnected [51,52]. The negative temperature rises when the intrinsic conductivity of the MWCNTs is dominated. As can be seen in Figure 11, the sensitivities of PDMS-covered specimens are similar to the uncovered specimens. The covered specimen with a filler content of 1.25 wt% shows a lower sensitivity, with 50% at 80 °C, than the uncovered specimen, with 54% at 80 °C. On the other hand, the covered PDMS specimen with a filler content of 2 wt% shows a higher sensitivity, with 18% at 80 °C, than the uncovered specimen, with 16% at 80 °C. The tendency of the humidity influence is not identifiable in this experiment. However, it has been proven that the temperature influence is clearly the dominant effect.

In contrast to the interconnected MWCNTs, the contact resistance and the tunnel resistance are responsible for the resistance change in a composite. The volume expansion of the epoxy in a 3D composite is more dominant and is responsible for the positive temperature coefficient. When the volume expands, the distances between the MWCNTs increases. This causes a higher tunneling resistance or the contacts between the MWCNTs will be broken up. The result is a rise of resistance by increasing temperature. The measured results correspond to the investigation of Lasater *et al.*, who have investigated the influence of the temperature on the resistance of CNT-epoxy composite [30].

**Figure 12:**
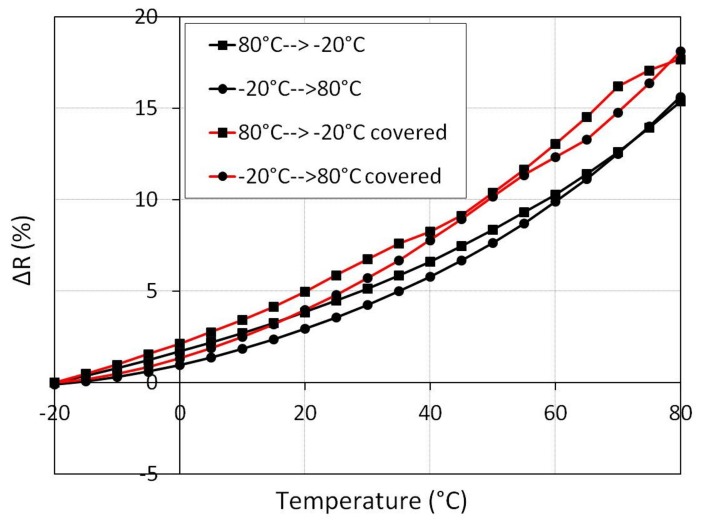
Resistance hysteresis in one temperature cycle (sample with 2 wt%). Black lines indicate the native MWCNT specimen; red lines indicate the PDMS-covered MWCNT specimen.

In order to investigate the reproducibility of the temperature influence, the resistance dependence of the cycle from −20 °C up to 80 °C is investigated in the next experiment. In the beginning, the temperature decreased from 80 °C to −20 °C. The value of the reference resistance at −20 °C is 3 kΩ. Afterwards, the temperature increased again to 80 °C. As can be observed in Figure 12, the resistance change of the specimen with 2 wt% before and after covering with PDMS shows a hysteresis.

The reason for the hysteresis could be the slow volume expansion/contraction processes of the epoxy. Nevertheless, the temperature dependency for one element is greater than the sensitivity to force and pressure. The whole resistance change for 2 kN is about 6% (see Figure 9) for a specimen with 2 wt%, while the resistance change caused by the temperature is 16% (Figure 12). This fact demonstrates that a pressure measurement using only one element is not recommended.

### 5.4. Temperature Compensation

**Figure 13:**
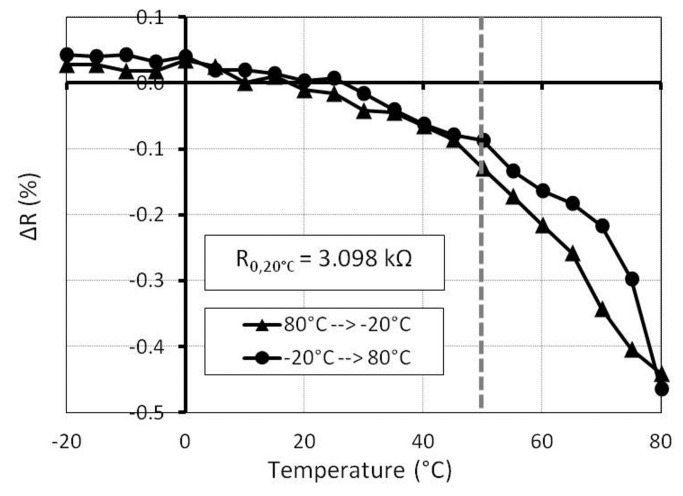
Temperature dependency of the Wheatstone bridge (sample with 2 wt%).

Temperature compensation is necessary because of the high temperature dependence of the MWCNT composite. The conditions for effective temperature compensation with a Wheatstone bridge are that all elements have the same temperature coefficient and the nominal resistance values are similar. The detailed working mechanism for the temperature compensation are explained in Section 3.1. Owing to the same material composition, the four elements have the same temperature coefficient. Additionally, the fabrication processes lead to a resistance variation of 3.1%. In this experiment, the same specimen with 2 wt%, like in the previous experiments, was used. Although all four elements are responsible for the temperature compensation (full bridge), the resistance is calculated by the Equation (1) for the Wheatstone bridge. This allows a calculation of an equivalent measurement error, which is caused by the temperature. The temperature response calculated by the half bridge equation is depicted in Figure 13.

In the whole temperature range from −20 °C−80 °C, the resistance change of the sensor structure is now only 1%. This is 16-times smaller than the temperature sensitivity of one element (see Figure 12). The negative temperature dependence of the resistance in this specimen is not applicable to the other specimens. Depending on the resistance distribution of the single element, the temperature dependence can have a positive or negative gradient. In other words, the positive or negative gradient is randomly distributed. The higher the similarity (*i.e.*, similar resistance value and temperature sensitivity) of the four elements, the higher is therefore the effectiveness of the temperature compensation.

When the working temperature is from −20 °C up to 80°C, the measurement error will be 550 N, which corresponds to 27.5% of the measurement range up to 2 kN. However, the working temperature range of most electronics is from −20 °C up to 50 °C. If this temperature range is considered, the resistance change in the bridge is only 0.3%. In this case, a measurement error of 150 N can be expected, which corresponds to 7.5% of in the measurement range up to 2 kN.

## 6. Conclusions

In this work, the force/pressure sensing behaviors of MWCNT-epoxy composite up to 2 kN (20 MPa) are investigated. For this aim, MWCNT-epoxy composite is dispersed first by a magnetic stirrer and second by a three-roll mixer. The resistance decrease after the three-roll mixing, leading to the conclusion that the MWCNTs are more efficiency dispersed. Hence, the viscosity is increased after the three-roll mixing process.

Additionally, this work reported the connection between pressure sensitivity, temperature dependency and electrical signal noise for a CNT composite. Pressure and temperature measurement show a higher sensitivity at lower MWCNT filler content. However, the electrical response of the lower filler content gives a noisy signal. For this reason, a compromise between these three aspects is needed for a sensor application. Samples with higher filler content (1.75 wt%, 2 wt%) show an improved total performance. At constant temperature, a force of at least 50 N (0.5 MPa) in a measurement range of 2 kN (20 MPa) can be resolved with a sensor structure of 2 wt%.

Temperature compensation was demonstrated with a Wheatstone bridge. Similar resistances are one condition for efficient temperature compensation. With the FEM modeling and the optimized fabrication processes, nominal resistances with a variation of 3.1% could be fabricated. By using a filler content of 2 wt%, it has been shown that temperature dependence is decreased from 16% to 1%. When the working temperature is defined from −20 °C−50 °C, the temperature influence leads to a measurement error of 150 N (1.5 MPa).
